# Complete mitochondrial genome of the hybrid of *Megalobrama terminalis*(♀) × *Culter alburnus*(♂)

**DOI:** 10.1080/23802359.2020.1716638

**Published:** 2020-01-24

**Authors:** Kai Liu, Heng-Jia Ma, Xiao-Yu Feng, Nan Xie

**Affiliations:** Institute of Fishery Science, Hangzhou Academy of Agricultural Sciences, Hangzhou, China

**Keywords:** Hybrid of *Megalobrama terminalis*(♀) × *Culter alburnus*(♂), mitochondrial genome, maternal inheritance

## Abstract

In this study, we determined the complete mitochondrial DNA sequence of the hybrid of *Megalobrama terminalis*(♀) × *Culter alburnus*(♂) for the first time. The complete mitochondrial genome of the hybrid was sequenced to be 16,621 bp in size following the female parent, *M. terminalis*. The genome contained 13 protein-coding genes, 22 transfer RNA genes, 2 ribosomal RNA genes, and 2 main non-coding regions (the control region and the origin of light strand replication). Sequence alignment between the mitochondrial genomes of the hybrid and its female parent showed that a total of 28 mutation sites were identified in 14 genes or regions. The genome information presented here may play an important role in further study on the genetic mechanisms of mitochondrial DNA in hybrids.

*Megalobrama terminalis* and *Culter alburnus* are two economically important fish species in the genus *Megalobrama* and *Culter*, respectively. *Megalobrama terminalis* mainly inhabited the middle and upper reaches of the Yangtze, Heilong River Basin, Qiantang River (Chen 1998). *C. alburnus* is widely distributed in major reservoirs, rivers, and lakes of China (Chen 1998). Owing to the good economic traits and strong adaptability, *C. alburnus* has been recognized as a main aquaculture species in China (Shi et al. 2017). *Megalobrama terminalis* has more delicious flesh than *C. alburnus*. In order to synthesize the important economic traits of the two freshwater fish, both collected from Qiantang River (120°10′13.15″E, 30°07′11.22″N), a population of the hybrid (assigned as SJFQZB201805) of *M. terminalis*(♀) × *C. alburnus*(♂) was obtained by artificial hybridization experiment, stored at national original breeding farm of Black Amur bream from Qiantang River (120°07′21.99″E, 30°08′35.53″N) and the complete mitochondrial genome in an individual of the hybrid F1 was sequenced.

Total genomic DNA of a specimen of the hybrid was extracted from the fin tissue using phenol-chloroform extraction method (Sambrook et al. 1989). 19 pairs of PCR primers were designed to amplify the whole mitogenome sequences based on the *M. terminalis* mitochondrial nucleotide sequences (GenBank No. MH289767). PCR products were directly sequenced by Personal Gene Technology CO., Ltd (Shanghai, China) after purification. The complete nucleotide sequence after assembled had been registered in the GenBank with the accession number MN604232. The annotation process was undertaken using MITOFISH prediction server (Iwasaki et al. 2013).

The phylogenetic tree of the hybrid of *M. terminalis*(♀) × *C. alburnus*(♂) is shown in Figure 1, which was drawn by the software MEGA X (Kumar et al. 2018). The length of the complete mitogenome sequence of the hybrid was 16,621 bp, similar to that of *M. terminalis*. The whole mitogenome contained 22 tRNA genes, 2 ribosomal RNA genes, 13 protein-coding genes (PCGs), and 2 main non-coding regions. Most of the genes of the hybrid were encoded on heavy strand (H-strand) except for ND6 and 8 tRNA genes, which were encoded on the light strand (L-strand). Within the genome, all the 13 PCGs included the orthodox start codon ATG except for COX1. However, the stop codons of the 13 PCGs were different with TAA, TA– or T–. The origin of light-strand replication (OL) which extends up to 32 nucleotides, was identified in a cluster of five tRNA genes (WANCY region) between tRNA-Asn and tRNA-Cys. The second non-coding region, the control region (D-loop), was located between the tRNA-Pro and tRNA-Phe genes with 937 bp in length.

**Figure 1.**
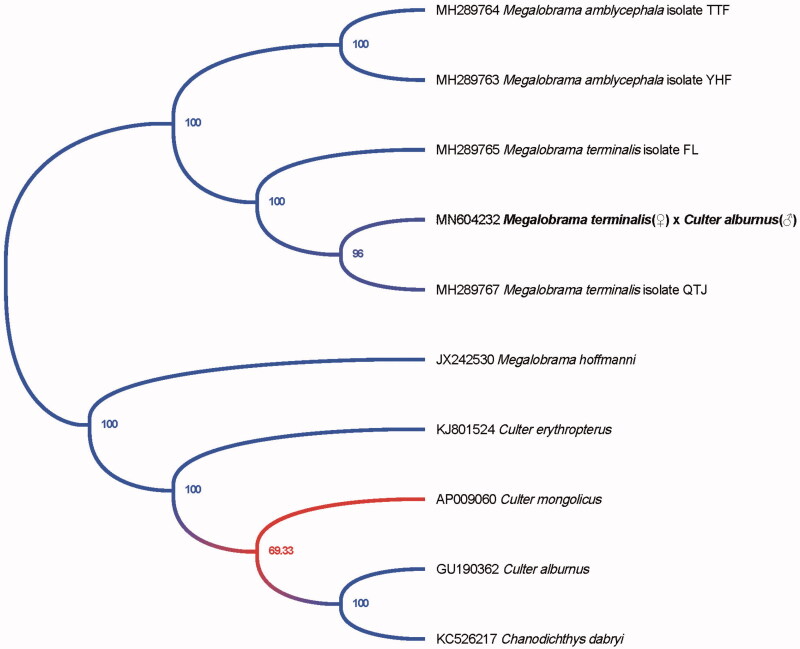
Phylogenetic tree of the hybrid of *M. terminalis*(♀) × *C. alburnus*(♂) and 9 species in the genus *Megalobrama* and *Culter* inferred by using the maximum likelihood (ML) method based on the complete mitochondrial genome data.

99.85% sequence identity between the hybrid and M. terminalis confirmed the maternal inheritance pattern followed by the mitogenome of the hybrid. However, a total of 28 mutation sites were found in 14 genes or regions of mitogenome of the hybrid.
